# Attention to the Registry of Neglected Diseases: Idiopathic Granulomatous Mastitis as an Example

**DOI:** 10.34172/ijhpm.2024.8697

**Published:** 2024-11-19

**Authors:** Sadaf Alipour, Mohammadreza Zafarghandi, Iran IGM Group

**Affiliations:** ^1^Breast Diseases Research Center (BDRC), Cancer Institute, Faculty of Medicine, Tehran University of Medical Sciences, Tehran, Iran.; ^2^Department of Surgery, Arash Women’s Hospital, School of Medicine, Tehran University of Medical Sciences, Tehran, Iran.; ^3^Sina Trauma and Surgery Research Center, Faculty of Medicine, Tehran University of Medical Sciences, Tehran, Iran.; ^4^Department of Vascular Surgery, Sina Hospital, Faculty of Medicine, Tehran University of Medical Sciences, Tehran, Iran.

## Dear Editor,

 Many diseases in the world are infrequent, and constitute less of a burden for global health than common conditions. For some of them, there is a rival disease that is very serious or common, and has drawn all the attention of researchers and health policy-makers. Consequently, the latter (disease B) inspires all studies, and the former (disease A) is rather neglected. While the logarithmic pace of scientific advances feeds and strengthens management guidelines for B, a paucity of knowledge occurs for A. In the long term, the gap between A and B enlarges too much and develops into a health disparity issue by depriving patients affected by A from optimal health. If the neglected A is a considerable source of morbidity, patients and physicians are confronted with an unanswered health challenge; whereas health authorities cannot turn the tide when administrative measures are not supplemented by original data.

 In these circumstances, providing a platform for the systematic record of information about A would be the first means toward modification of the imbalance. Development of a registry dedicated to A and its implementation over an extended geographical area will gradually enable a methodical evaluation of the disease at a low cost.

 However, surveillance and registry of rare diseases face some challenges: First, the funding organizations usually invest on more common diseases. Second, the registry should be catered by many centers to collect enough data, while that rare disease is not their main concern. Third, the lack of familiarity of physicians may cause underdiagnosis and under-reporting of the disease.

 Different levels of public health management can help mitigate these issues. Governments could play a critical role by ensuring sustainable funding and motivating accurate disease report. The development of specialized centers at national levels that would serve as the core for training health professionals and directing studies could upgrade diagnosis and reporting. The coordination of regional and local centers with the core center, recurrent training of their health staff, and accurate data record would complement the process.

 One of the neglected diseases worldwide is idiopathic granulomatous mastitis (IGM). Although published studies implicate a higher frequency of the disease in Turkey, Iran, China, India, and in the American Hispanics,^1^ on a global account, IGM is a rare disease of the breast. However, IGM or disease A is behind a large B, because the breast is home to breast cancer, the most common female malignancy worldwide, and the number of studies around it is massive; thrusting IGM in the dark. Now it comes that although not malignant, IGM is associated with significant morbidity due to its chronicity, recurrences, disturbing presentation, and irreversible deformities.^2^ Despite its recognition in 1972,^3^ the epidemiology, risk factors, best diagnostic, and treatment approaches are still unknown.

 Considering the data deficiency about this disease, we designed a project to develop and launch a hospital-based (medical center-based) registry for IGM (RIGM) (Ethical Approval from the Ethics Committee of Tehran University of Medical Sciences: IR.TUMS.SINAHOSPITAL.REC.1401.063). After an all-inclusive search of the literature and several expert meetings, the Minimum Dataset (Supplementary file 1) was defined. Then the RIGM software was developed as a user-friendly short tool with an English interface to permit international collaboration.

 So far, 352 patients have been recorded in RIGM, and 4 centers have signed collaborating agreements while 10 others are considering it; so RIGM will constitute a multicentric study. It is directed by the IGM (core) Clinic of the Breast Diseases Research Center of Tehran University of Medical Sciences. The plan is the future collaboration of all members of the Iran IGM group, plus international collaborations.

 We faced some obstacles including convincing higher authorities about the necessity of the project, foreseeing the financial aspects, calling on experts to bring them into play despite their concurrent activities in breast cancer studies, applying quality control, and attracting national and international collaborators.

 For other diseases ignored behind more frequent conditions, launch of global dedicated registries would be the best first policy on the road to health fairness. However, the registry of neglected diseases is also being neglected; scientists and health managers should care about it.

## Acknowledgements

 We would like to acknowledge the collaborators in the Iran IGM Group (in alphabetical order: Abasvandi, Fereshteh; Assarian, Abdolali; Ahmadi, Najmeh; Aghajani, Reyhaneh; Aziminezhadan, Parisa; Astaraki, Shahla; Bolourinejad, Paria; Borjian, Anahita; Dehghani, Narges; Dabbagh, Najmeh; Eslami, Bita; Famil-Amir, Paniz; Fattahi, Asieh S.; Farbod, Meraj; GhariniAhmadi, Maryam; Hakimian, Mohammadreza S.; Hashemi, Esmat S.; Hashemian, Maria; Hessamiazar, Shiller; Hosseinpour, Reza; Jabbari-Nooghabi, Azadeh; Jalili, Roghayeh; Javadi, Mohammadreza S.; Joulaee, Azadeh; Khajeh-Ali-Beiki, Behnaz; Madani, Fakhrolsadat; Mahmoodzadeh, Habibollah; Mashoori, Negar; Mazinani, Azita; Molavi, Sima; Nafissi, Nahid; Najafi, Safa; Noori, Matina; Olfatbakhsh, Asieh; Omranipour, Ramesh; Orouji, Marzieh; Saberi, Azin; Salati, Azam; Sarkardeh, Maryam; Tabatabaeian, Maryam; Tahery-Mehr, Reihane; Tavakol, Mahsa; Vardkar, Ziba; Zahernia-Shahrbabaki, Zohreh; Ziaee, Fataneh) for their collaboration in this study, and the Anahid Clinic (Isfahan, Iran) for their support in all studies related to IGM.

## Ethical issues

 This study has been approved by the Ethics Committee of Tehran University of Medial Sciences, Tehran, Iran (Ethics Code: IR.TUMS.SINAHOSPITAL.REC.1401.063).

## Conflicts of interest

 Authors declare that they have no conflicts of interest.

## Supplementary files

Supplementary file 1 contains Table S1.
