# Supplementary material for: Attention to the Registry of Neglected Diseases: Idiopathic Granulomatous Mastitis as an Example
Source: Int J Health Policy Manag. 2024 Nov 19;13:8697. doi: 10.34172/ijhpm.2024.8697 (PMC11607736; doi:10.34172/ijhpm.2024.8697)
Supplement: Supplementary file 1 — contains Table S1. [file ijhpm-13-8697-s001.pdf]

Article title: Attention to the Registry of Neglected Diseases: Idiopathic Granulomatous Mastitis as an Example

Journal name: International Journal of Health Policy and Management (IJHPM)

Authors' information: Sadaf Alipour[1,2]*, Mohammadreza Zafarghandi[3,4], Iran IGM Group[1]

[1]Breast Diseases Research Center (BDRC), Cancer Institute, Faculty of Medicine, Tehran University of Medical Sciences, Tehran, Iran.

[2]Department of Surgery, Arash Women's Hospital, School of Medicine, Tehran University of Medical Sciences, Tehran, Iran.

[3]Sina Trauma and Surgery Research Center, Faculty of Medicine, Tehran University of Medical Sciences, Tehran, Iran.

[4]Department of Vascular Surgery, Sina Hospital, Faculty of Medicine, Tehran University of Medical Sciences, Tehran, Iran.

***Correspondence to:** Sadaf Alipour; Email: sadafalipour@yahoo.com

Citation: Alipour S, Zafarghandi M, Iran IGM Group. Attention to the registry of neglected diseases: idiopathic granulomatous mastitis as an example. Int J Health Policy Manag. 2024;13:8697. doi:10.34172/ijhpm.2024.8697

Supplementary file 1

Supplementary Table. Minimum dataset of Idiopathic Granulomatous Mastitis Registry

**SECTION1: GENERAL FORM**
GENERAL ITEMS **Date of filing:** ...../...../.......... (day / month/ year) **Patient's first name** (in English): …………… **Patient's Last name** (in English): ……………… **Patient's first name** (in local language): ……………… **Patient's Last name** (in local language): ……………… **Father's Name**: …………………… **National code number:** ………………… **Date of Birth:** ……………… **Phone number1:** ..…...........… **Phone number2:** ….............. **Responsible physician:** ……… **Specialty:** …………… **Name of facility:** …………… **City of practice:** …………… **Province/state of practice:** ………… **Country of practice:** ……………
DEMOGRAPHICS, GEOGRAPHICS, HABITS **Age**… years **Sex** Female☐ Male☐ **Weight**…kg **Height**…cm **Personal Ethnicity** (*Defined for each country)* **Place of Birth (*in local language*)** Province/State……........… City/Village…….. **Place of Residency (*in local language*)** Province………….. City/ Village…………..

Hookah (water pipe) use Unknown ☐ Yes ☐ No ☐ **Smoking** Unknown☐ Current smoker☐ Former smoker☐ Fancy smoker☐ Passive smoker☐ Never ☐

REPRODUCTIVE FEATURES **Age at menarche** ……years old Unknown ☐ **Menopausal status** Unknown ☐ Menopause ☐ Premenopausal ☐

Any history of pregnancy Unknown☐ No☐ Yes☐ *If yes,* **Currently pregnant** No☐ Yes☐

Gravidity… **Parity**… **Abortion**… **Time since last delivery**…months

History of breast feeding Unknown☐ No☐ Yes☐ *If yes,* **Currently breastfeeding** No☐ Yes☐

Total breastfeeding …months **Time since last breastfeeding** …months

Side of breastfeeding Unknown ☐ Both equally ☐ Only left ☐ Only right ☐ Left more ☐ Right more ☐

History of OCP use Unknown ☐ No ☐ Yes ☐ *If yes,* **Duration of OCP use**…….months

Time since last OCP ………months

PAST MEDICAL HISTORY **Family history of autoimmune or rheumatologic disease (1st degree)** Unknown ☐ No ☐ Yes ☐

Any disease Unknown☐ None☐ Autoimmune☐ DM☐ HyperPRLemia☐ HTN☐ Hyperthyroidism☐ Hypothyroidism☐ Psychiatric☐ TB☐ Others☐ *please specify*…

Self-drug history, other than IGM Unknown☐ None☐ Anti-TB☐ Diabetic☐ Immunosuppressives☐ Levothyroxin☐ Psychiatric Others ☐ *please specify*…...

PREVIOUS IGM **History of previous IGM** Unknown☐ No☐ Yes☐ *If yes,* **How many recurrences**… **Side of previous IGM** Unknown☐ Left☐ Right☐ Bilateral☐

Time from recurrence……months **Previous treatments** Unknown☐ Medical☐ Observation☐ Surgery☐ Traditional☐ *please specify*…… **Medicines** *IN THE SYSTEM* **Surgery** Unknown☐ None☐ Incision.&drainage☐ Lumpectomy☐ WLE☐ Mastectomy☐ Mast.&reconstruction ☐ Others, *please specify* ……

SECTION 2: ACTIVE IGM FORM

CLINICAL PRESENTATION **Start of current presentation** ……days ago **Season** Autumn☐ Spring☐ Summer☐ Winter☐ **Side** Left☐ Right ☐ Bilateral ☐

Presenting symptoms Unknown☐ Abscess☐ *Number* …… *Size of the largest...mm* Arthralgia☐ Chills☐ Edema☐ Erythema☐ Fever☐ Fistula ☐*Number*….

Itching☐ LAP☐ Mass/Induration☐ *Number …. Size of the largest ….. mm* Nipple discharge☐ Nipple retraction☐ Orange peel-like skin changes☐ Pain/tenderness ☐ Ulcer ☐ *Number …… Size of the largest…..mm* Others, *please specify……….*

IMAGING FINDINGS **Mammography performed** Unknown ☐ No ☐ Yes ☐ *If yes,* **Date**………………… **Center name (*in local language*)**………………

Mammography BIRADS Unknown☐ B0☐ B1☐ B2☐ B3☐ B4a☐ B4b☐ B4c☐ B4any☐ B5☐ B6☐ **Mammographic density** Unknown☐ A☐ B☐ C☐ D☐

Ultrasound performed No☐ Yes☐ *If yes,* **Date**………**Center name**……………….**Ultrasound BIRADS** Unknown☐ B1☐ B2☐ B3☐ B4a☐ B4b☐ B4c☐ B4any☐ B5☐ B6☐

Ultrasound findings Unknown☐ Normal☐ Collection☐ Distortion☐ Edema☐ LAP *No☐ Normal/Reactive☐ Suspicious☐* Mass☐ *multiple☐ solitary☐*

Margin *Circumscribed☐ Non-circumscribed (including indistinct, angular, microlobulated, spiculated)☐* Orientation *Non-parallel/vertical☐ Parallel/horizontal☐*

Shape *Irregular☐ Round/oval☐* Increased vascularity *Unknown☐ No☐ Yes☐* Non-mass lesion ☐ Sinus tracts ☐

MRI performed Unknown☐ No☐ Yes☐ *If yes,* **Date**…………… **Center name**…………………. **MRI BIRADS** B1☐ B2☐ B3☐ B4☐ B5☐ B6☐

LABORATORY TEST **Blood tests performed** Unknown☐ No☐ Yes☐ *If yes,* **Date**……… **Center name**………… **ESR** … **CRP** Unknown☐ Neg☐ Pos☐..........

CBC Hb ….. WBC …........... Neutrophil …..% Lymphocyte ….% Eosinophyl ...% Monocyte ….%

TSH….. **PPD**….. **Prolactin (unit)** ….. (.....) **Vitamin D**…..

Blood Group&Rh Unknown☐ A+☐ A-☐ B+☐ B-☐ AB+☐ AB-☐ O+☐ O- ☐

Microorganism assessment performed Unknown☐ No☐ Yes☐ *If yes,* **Date**……… **Center name**………………….

Type of assessment Unknown☐ Culture☐ *one time☐ several times☐* PCR☐ *one time☐ several times☐* Smear☐ *one time☐ several times☐*

Type of microorganism detected Unknown☐ None☐ Mycobacteria(Acid-fast bacilli)☐ *Please specify* type ……………….. Other ☐ *Please specify* type ………………..

HISTOLOGY **Type of biopsy** Unknown☐ CNB☐ VAB☐ Surgery☐ Other ☐ *please specify* ………………….....

Histology report available No☐ Yes☐ *If yes,* **Date**……….. **Center name**……………………. **Reference Number**……………………

Findings: Abscess None☐ Eosinophilic Abscesses: *Microabscess☐ Macroabscess☐*
Neutrophilic abscesses: *Microabscess☐ Macroabscess☐*

Cholesterol crystals No☐ Yes☐ Duct ectasia No☐ Yes☐ Fat necrosis No☐ Yes☐

Inflammation pattern None☐ Lobulocentric☐ Non-Lobulocentric☐ Other☐

Granulomas Uncertain☐ No☐ Yes☐ *If yes, Necrotizing☐ Non- necrotizing☐ Poorly formed☐ Sarcoidosis-like☐*

Infiltration None☐ Eosinophil-rich☐ Histiocyte-rich☐ Lymphocyte-rich☐ Neutrophil-rich☐ Plasma cell-rich☐ Other☐

Multinucleated giant cells None ☐ Combined types☐ Foreign body☐ Langhans-type☐

Interpretation GM (GLM/ IGM) ☐ Cystic neutrophilic GM☐ Mastitis NOS☐

Comment...

FIRST-LINE VISIT AT OUTSIDE FACILITY **Diagnosed at outside facility** Unknown☐ No☐ Yes☐ *If yes,***Name of facility**……… **Time from first visit at outside facility**….days

Types of treatments Unknown☐ Medical treatment☐ Observation ☐ Surgery (*Listed later*)☐ Traditional methods ☐ *please specify*………………….

Medicines used at outside facility *IN THE SYSTEM* **ABs used at outside facility** *IN THE SYSTEM*

Surgery at outside facility Unknown☐ None☐ I&D☐ Partial excision☐ Complete excision☐ WLE☐ Mastectomy☐ Mast.&reconstruction☐ Others, *please specify*..........

Response to treatment at outside facility Unknown☐ Improved☐ Resolved☐ No change☐ Worsened☐ **Overall duration of treatment at outside facility**……weeks

Reason for attending your facility Unknown☐ Consultation☐ Follow-up☐ No response to treatment☐ Referred from the previous facility ☐ Other ☐

TREATMENT AT YOUR FACILITY **Date of first visit at your facility**………………………

Types of treatments Unknown☐ None☐ Medical☐ Observation☐ Surgery☐ Traditional☐*please specify*………… **Medicines** *IN THE SYSTEM* **ABs** *IN THE SYSTEM*

Type of Surgery Unknown☐ None☐ I&D☐ Partial excision☐ Complete excision☐ WLE☐ Mastectomy☐ Mast.&reconstruction☐ Others, *please specify*...........

**Response to: Observation** NA☐ Unknown☐ Resolved☐ Improved☐ Same☐ Worsened☐<br>**Traditional** NA☐ Unknown☐ Resolved☐ Improved☐ Same☐ Worsened☐<br>**Medical** NA☐ Unknown☐ Resolved☐ Improved☐ Same☐ Worsened☐ **Surgical** NA☐ Unknown☐ Resolved☐ Improved☐ Same☐ Worsened☐<br>**Duration of treatment at your facility**………...weeks **Time from presentation to resolution/stability**………...weeks
ADVERSE DRUG REACTIONS **Occurrence of adverse drug reactions** No☐ Yes☐ *If yes,* **Name of medicines**…..........…. **Type of adverse reaction**………......
**SECTION3: 3-, 6-, 12, 24 MONTHS FOLLOW-UP**
**Date**…………… **Symptoms** Unknown☐ Resolved☐ Improved☐ Stable☐ Worsened☐<br>**Cosmesis** Unknown☐ Improved☐ Stable☐ Worsened☐<br>**Continuation of treatments in the interval** Unknown☐ No☐ Yes☐ *If yes,* **Types of medicines in the interval** *IN THE SYSTEM*<br>**Received new treatments in the interval** Unknown☐ No☐ Yes☐ *If yes,* **Types of treatments** Medical☐ Observation☐ Surgery☐ Traditional☐*please specify*............<br>**Medicines used in the interval** *IN THE SYSTEM* **ABs used in the interval** *IN THE SYSTEM*<br>**Surgery in the interval** Unknown☐None☐ I&D☐Partial excision☐Complete excision☐WLE☐ Mastectomy☐ Mast.&reconstruction☐ Others, *please specify*..........<br>**Response to treatment** Unknown ☐ Improved☐ Resolved☐ Stable☐ Worsened ☐<br>**Duration of treatment**………...weeks

The table has been slightly modified for the article. For all medications, the method of administration including topical, oral, local injection and systemic injection as well as the administered daily dose and the duration of treatment are asked. DM= diabetes mellitus, HTN= hypertension, I&D= Incision and Drainage, IGM= Idiopathic granulomatous mastitis, LAP= lymphadenopathy, NA= Not applicable, Neg= negative, Pos= positive, PRL= prolactin, TB= tuberculosis, WLE= wide local excision.
